# PRO-Angoff method for remote standard setting: establishing clinical thresholds for the upper digestive disease tool

**DOI:** 10.1186/s41687-024-00707-x

**Published:** 2024-03-12

**Authors:** Minji K. Lee, Mohamad K Abou Chaar, Shanda H Blackmon, Kathleen J Yost

**Affiliations:** 1 Department of Quantitative Health Sciences, Mayo Clinic, 200 1st Ave SW, 55902 Rochester, MN USA; 2 Department of Surgery, Division of Thoracic Surgery, Mayo Clinic, Rochester, MN USA

**Keywords:** Remote monitoring of symptoms, Upper digestive disease, Standard setting, Cut score, Angoff method

## Abstract

**Background:**

The Upper Digestive Disease (UDD) Tool™ is used to monitor symptom frequency, intensity, and interference across nine symptom domains and includes two Patient-Reported Outcome Measurement Information System (PROMIS) domains assessing physical and mental health. This study aimed to establish cut scores for updated symptom domains through standard setting exercises and evaluate the effectiveness and acceptability of virtual standard setting.

**Methods:**

The extended Angoff method was employed to determine cut scores. Subject matter experts refined performance descriptions for symptom control categories and achieved consensus. Domains were categorized into good, moderate, and poor symptom control. Two cut scores were established, differentiating good vs. moderate and moderate vs. poor. Panelists estimated average scores for 100 borderline patients per item. Cut scores were computed based on the sum of the average ratings for individual questions, converted to 0-100 scale.

**Results:**

Performance descriptions were refined. Panelists discussed that interpretation of the scores should take into account the timing of symptoms after surgery and patient populations, and the importance of items asking symptom frequency, severity, and interference with daily life. The good/moderate cut scores ranged from 21.3 to 35.0 (mean 28.6, SD 3.6) across domains, and moderate/poor ranged from 47.5 to 71.3 (mean 54.5, SD 7.0).

**Conclusions:**

Panelists were confident in the virtual standard setting process, expecting valid cut scores. Future studies can further validate the cut scores using patient perspectives and collect patient and physician preferences for displaying contextual items on patient- and physician-facing dashboard.

**Supplementary Information:**

The online version contains supplementary material available at 10.1186/s41687-024-00707-x.

## Introduction

Routine remote monitoring of symptoms and morbidity among high-risk patients improves patient-centered care and can lead to improved outcomes [1]. In our prior work [2, 3], we developed and validated the CONDUIT questionnaire, which measures five multi-item domains for monitoring patient function and well-being after esophageal reconstruction. Working with a panel of experts in esophageal reconstruction, we conducted a standard setting exercise to establish clinical thresholds for CONDUIT domain scores to categorize patients post-reconstruction into three clinically meaningful groups of “good” (no action; green), “moderate” (patient education on symptom self-management; yellow), and “poor” (care team contact; red) [3]. We collaborated with the Mayo Clinic Center for Connected Care to develop an app based on the CONDUIT tool, with automatic score reporting and color-coded severity communication to facilitate interpretation by the clinical team [4, 5]. Given success of the project, we recently expanded the tool, which was renamed the Upper Digestive Disease (UDD) Tool™ to better reflect the expanded content and intended population beyond esophagectomy to foregut reconstruction including gastrectomy, pancreatectomy, bariatric, and possibly other diseases. The UDD Tool™ is available as an app and paper format. As a result of the changes to the tool, we needed to update clinical thresholds.

The two aims of this study were to (1) conduct a standard-setting exercise to establish thresholds for the updated domains for the UDD Tool™; (2) assess panelist perceptions of engaging in a virtual standard setting meeting.

## Methods

### The UDD tool™

The UDD App is an electronic patient-reported outcome measure (PROM) and symptom-reporting and -monitoring tool that includes nine symptom domains (dysphagia, pain, dyspnea, aspiration, heartburn, regurgitation, nausea, and two dumping syndrome domains). It also administers two PROMIS (Patient-Reported Outcome Measurement Information System) Global Health domains: Physical and Mental Health. If a patient indicates having “never” experienced the symptom in the given recall period, the tool navigates the patient to the next domain. There are 76 items in total currently. Domain scores are reported on a 0-100 scale with higher scores indicating worse symptom control. The UDD questionnaire is accessible in both digital and paper format, collectively referred to as the UDD Tool™. UDD App is copyrighted by Mayo Foundation for Medical Education and Research, is available to all people free of charge, and can be used for not-for-profit clinical care or research.

This tool is intended to be used no sooner than 30 days after the surgery for esophagectomy patients but possibly sooner for other foregut procedures. Repeated assessments are recommended every 90 days during the first-year post-surgery, bi-annually for the second year, and then once a year thereafter indefinitely [4].

### Rationale for standard setting

As with many PROMs, interpretation of UDD Tool™ domain scores on a 0-100 scale is not intuitive, which makes it hard for healthcare providers to use the information in clinical practice. One way to facilitate interpretation and actionability of UDD Tool™ scores is to set specific score threshold to classify patients into clinically relevant groups. Methods for establishing score thresholds (cut scores) for screening PROMs include (1) normative values, (2) criterion validity, and (3) standard setting. Normative values require a large pool of data, which is not yet available for the UDD Tool™. Criterion validity requires a “gold standard” or external criterion for identifying clinically relevant groups; external criteria are not currently available for UDD Tool™ domains. Standard setting methods rely on stakeholder engagement to establish score thresholds. Through an iterative process, subject matter experts (SMEs) on the patient population identify the range of PRO scores that will define clinically actionable levels of symptom severity or functional limitations.

### Participants

We convened a nationwide panel of UDD patient care experts and stakeholders, including medical doctors and advanced practice providers. They developed the performance descriptions or completed rating tasks. Their participation was voluntary, and we renumerated the SMEs who took part in the rating tasks with a token gift (wooden letter box to store letters from grateful patients) valued at approximately $35.

### Performance description for standard setting

A prerequisite step to establishing cut scores is to develop accurate performance description for reference during rating. We determined three patient management categories: Good, moderate, and poor. The existing performance descriptions [3] for each category (i.e., good, moderate, poor) were refined, obtaining consensus from 16 stakeholders via iterative virtual communications.

### Standard setting meetings via video conferencing

Standard setting was conducted virtually via video conferencing, enabling us to engage experts from multiple institutions. Fifteen diverse stakeholders of medical doctors and advanced practice providers comprised the panel for the virtual standard setting meetings, who provided ratings, and achieved consensus on item-level cut scores. There was an interim evaluation meeting to test how the cut score-based decisions matched physician judgments regarding patient performance based on responses.

Before each virtual standard setting exercise, we sent training materials via email. At the beginning of each meeting, we reviewed key concepts (e.g., definition of borderline patients, rating methods) and answered any questions before beginning the rating tasks.

**Fig. 1:**
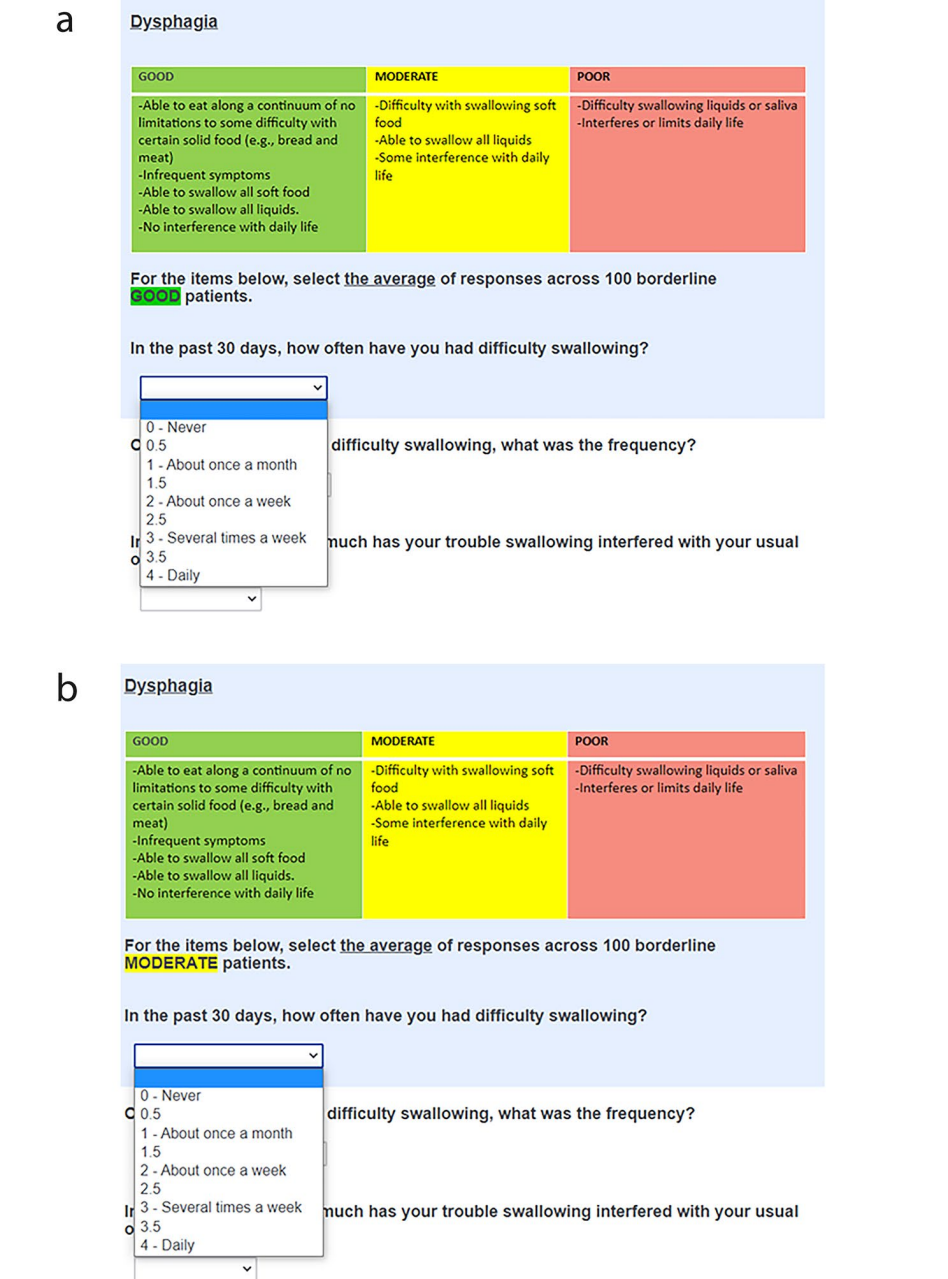
Excerpts from the Qualtrics web-survey. **(a)** The rating task for the borderline good patient. **(b)** The rating task for the borderline moderate patient

To establish two cut scores dividing patients into “good” and “moderate,” and “moderate” and “poor” categories, we asked panelists to consider both groups of borderline patients. Using a Qualtrics web-survey, each panelist read the general performance descriptions and the performance descriptions for each specific domain. Then, within the web-survey link (Fig. 1a), they estimated the responses that would be given by borderline good patients (i.e., the worst performing within the good category). The process was repeated for borderline moderate patients (i.e., the worst performing within the moderate category) as shown in Fig. 1b. Descriptive statistics of panelists’ ratings were computed in real time and displayed via the moderator’s shared screen. Using the “retake survey” function in Qualtrics, panelists were able to adjust initial ratings following group discussion.

#### Standard setting method

We used the extended Angoff standard setting method [6–8], originally developed in educational testing context, and then was adapted to health services research as in this study. The application of this method within the context of PROM is also detailed in our prior publication [3]. In the rating session, panelists estimated the average score that borderline patients would obtain on an item. For example, as shown in Fig. 1a, each panelist used drop-down options to mark how borderline good patients would respond to an item. To accommodate finer distinctions, we permitted the selection of half points between response options, enabling panelists to indicate values that fall between the given choices. For example, one panelist might assess that the least well-performing good patients would report difficulty swallowing approximately once a month, selecting a score of ‘1’. Another panelist, interpreting the description of worst performing good patients as having symptoms less frequently than once a month, might opt for a score of 0.5. Meanwhile, a third panelist could conclude that the frequency of symptoms falls between about once a month (1) and about once a week (2) for borderline good patients, and therefore choose a score of 1.5. This process was applied for moderate/poor cut scores (Fig. 1b), with panelists selecting higher scores than for borderline good cut scores.

Panelists individually estimated the average response borderline patients might select for each item, with the aggregate of these item-level estimates by a panelist forming their proposed cut score. The overall cut score was then determined by averaging these individual cut scores across panelists. We calculated the standard error (SE) of the cut score by dividing the standard deviation of the cut scores by the square root of the number of panelists. Subsequently, both the cut scores and their SEs were transformed to a 0-100 scale for reporting purposes.

#### Standard setting procedure

***Rating sessions***. There were several sessions for panelists to attend. Panelists engaged in at least one 90-minute sessions. In Round 1, panelists submitted initial ratings, followed by in-depth discussions. Round 2 allowed them to adjust ratings.

**Fig. 2:**
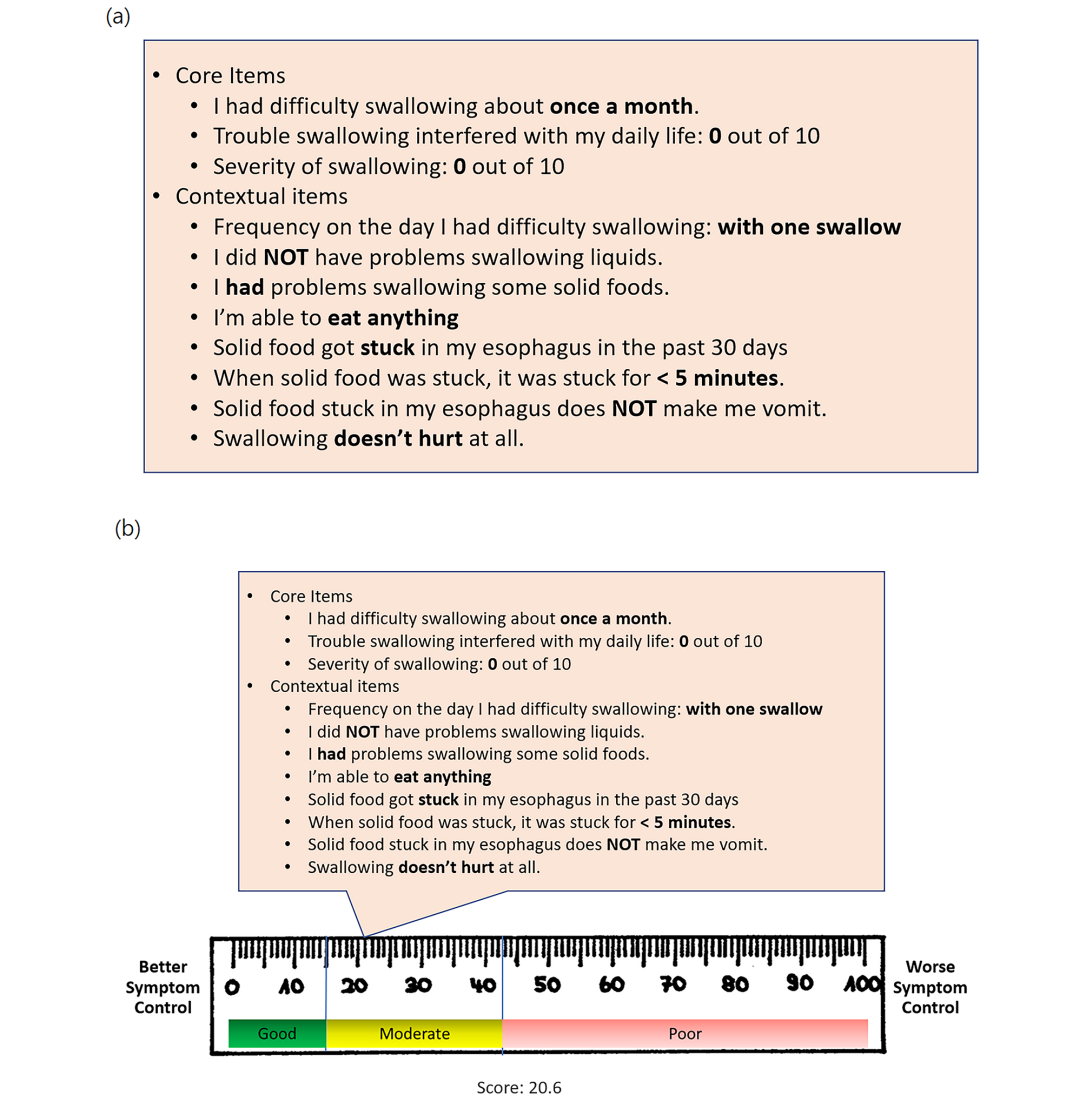
Excerpts for the interim evaluation meeting. **(a)** Actual responses of a patient. **(b)** Category assigned by the interim cut scores

***Interim evaluation meeting***. We held an interim meeting, where we assessed the validity of categorizing patients into three groups based on the interim cut scores derived from panelists’ ratings in the sessions up to that point (as depicted in Fig.2 ).

We provided responses from selected patients who completed the tool and asked panelists to deduce their performance category based solely on these responses. Subsequently, we unveiled the patient categorization using the interim cut scores. This sparked discussions among panelists regarding their level of agreement with these cut scores.

#### Final procedure

We held remaining sessions applying recommendations from the SMEs in the interim evaluation. Cut scores were derived and the SEs of the cut scores were computed.

## Results

### Performance description development

SMEs produced the performance descriptions iteratively in three separate meetings. The final performance descriptions are in Table 1. The SMEs also developed symptom-specific performance descriptions. Panelists also discussed that for weight, evaluation should be based on goals set by nutritionists and the expected trajectory for different surgeries (e.g., esophagectomy, pancreatectomy, achalasia).

### Standard setting discussions

Panelists provided their ratings with a focus on post-esophagectomy patients. The discussion between the Round 1 and Round 2 ratings focused on shifts in performance category interpretation over time, the core scoring elements, the relevance of medication in scoring, and generalizing standard setting results to various UDD patients.

***Time since surgery***. Panelists observed that the perception of the same symptom could differ based on the time elapsed since surgery. Frequent pain shortly after surgery might be anticipated and would not warrant an educational or clinical intervention, but the same frequency six months post-surgery might warrant an intervention. They concurred that implementing distinct cut scores for varying timepoints wasn’t advisable, maintaining the definitions of performance categories consistently across timepoints. They also favored combining performance categories with timepoints to guide decisions regarding when a patient should seek medical attention.

***Core Items.*** The distinction between “core” versus “contextual” items emerged during the interim evaluation meeting (Appendix 1). While most decisions aligned between the cut scores and panelists’ categorizations, there were instances where discrepancies arose. For example, for the responses shown in Fig. 2a, panelists categorized the patient as “good”, whereas the interim cut scores categorized this patient as “moderate” (Fig. 2b). The initial interim cut scores, which were based on both core and several contextual items, established a good/moderate cut score at 15 and a moderate/poor cut score at 42.5 for dysphagia, as illustrated in Fig. 2b. Our earlier cut scores, as reported in Lee et al. [3] similarly integrated both core and multiple contextual items.

Panelists contended that this patient shown in Fig. 2a and b should be deemed “good,” based on patient’s dysphagia symptoms manifesting only once a month (core item), and the absence of interference with daily life or symptom severity (core item). Other questions such as whether the patient had a dilation for their dysphagia was considered important contextually for interpreting the domain score and deciding on clinical action. Core items are measured on a 5-point ordinal rating scale (frequency questions) or a 0–10 numeric rating scale (severity and interference questions), whereas the response scales for the contextual questions vary, often being binary. Combining the contextual questions with the core questions would entail applying weights to many of them. For example, answering “yes” to one item may require more weight compared to another item if it implies more problematic symptom, but determining that weighting was considered too complicated and would decrease the transparency of the scoring for users of the tool.

During this gathering, the panelists concurred in a general sense that while all information held relevance, they would prioritize the items of frequency, severity, and interference. As a result of these discussions, the study team concluded that domain scores would henceforth be solely based on these three core items with contextual items presented as ancillary information alongside the scores.

***Medication***. Attention was drawn to the medication-related items across the questionnaire. Panelists pointed out the variability in medication efficacy and its clinical significance. What works for one patient might not for another. Within a patient, the medication may work for one day but not another. Given these intricacies, panelists advocated for maintaining the symptom assessment, independent of medication or therapy inputs.

***Dumping syndrome domains.*** Dumping syndrome is a condition that can occur after surgical procedures that remove or bypass the stomach such as an esophagectomy. This syndrome is characterized by a group of symptoms that result from the rapid emptying of stomach contents into small intestine. The manifestation of dumping syndrome symptoms is known to be highly variable among patients and the difficulties in its diagnosis and management are well-documented [9]. Aligning with these challenges, our previous study [10] highlighted discrepancies between the patient-reported dumping-related symptoms using the UDD tool and evaluations conducted by healthcare providers. In the current study, the SMEs assessed content relevance of the items in the dumping domains within the UDD tool and recommended the exclusion of certain term or items such as “shock” from the item, “shock which may involve becoming pale, having a weak pulse, or a very low blood pressure,” due to the term’s severe connotation. They reached agreement on removing “restlessness” and “breathlessness” from the dumping-generalized domain due to their content irrelevance. Additionally, symptoms like nausea, belching, and burping were considered non-indicative of dumping-gastrointestinaI (dumping-GI) symptoms, and recommended for removal from the questionnaire.

**Other suggestions.** SMEs noted the importance of dysphagia timing during the eating cycle—beginning, throughout a meal, or end—as it offers insights into potential causes (e.g., overeating, stricture). For example, dysphagia at the end of a meal end could stem from overeating, prompting behavior modification, while dysphagia at the meal’s onset might signal stricture. Essentially, the timing information guides care pathway such as dilation or behavior modification.

### Final cut scores

Based on panelist feedback, we investigated how much internal consistency estimates (α) changed if we only included core items for scoring. Using 50 surveys collected prior to standard setting study and with only the core items included, the α’s were above 0.80 for all domains except for dumping-GI (Table 2). For dumping-GI, the α was only 0.55. The dumping-GI frequency item had a low item-total correlation, and removing it increased α to around 0.70. Therefore, for dumping-GI, the scoring algorithm was tentatively adjusted to only include severity and interference items, and the frequency item will be treated as a contextual item.

**Table 1:** Performance descriptions for UDD symptoms

	Good	Moderate	Poor
Overall	Patients are performing as expected, and do not require an intervention or evaluation. Can continue to surveillance.	Patients not performing as expected. They may need medical attention over time. Patients would receive targeted education and counseling about behavior and diet followed by re-assessment.	Frequent or severe symptoms. Requires evaluation and may need an intervention
Dysphagia	• Able to eat along a continuum of no limitations to some difficulty with certain hard solid food (e.g., bread and meat) • Infrequent symptoms • Able to swallow all soft food • Able to swallow all liquids • No interference with daily life	• Difficulty with swallowing soft food • Able to swallow all liquids • Some interference with daily life	• Difficulty swallowing liquids or saliva • Interferes or limits daily life
Heartburn	• No burning • No symptoms when sleeping with the head elevated • No symptoms with or without medication • No interference with daily life	• Elevating the head of the bed helps but not completely • Tolerable symptoms persist despite medication. • Aware of the symptoms but easily tolerated • Some interference with daily life	• Aspiration • Constant symptoms • Severe symptoms persist despite medication • Severe burning symptoms when sleeping with head elevated • Burning feeling in throat or behind breastbone that interferes with activities or daily life • Interferes or limits daily life
Regurgitation	• No symptoms • No food or liquid moving in the wrong direction • No interference with daily life	• Occasional food or liquid washes up or returns to the mouth or throat • With medication, symptoms may be relieved • Lack of spontaneous regurgitation (identifiable triggers) • Predictable symptoms • Related to postural position (bending over, lying flat) or volume of food • Some interference with daily life	• Choking from fluid or ingested food that comes back to the mouth or throat • Daily occurrence • Poorly tolerated spontaneous regurgitation without identifiable triggers • Symptoms persist in spite of sitting upright or not eating before bed • Interferes or limits daily life
Dumping - Generalized	• Experiences no or minor symptoms^1^ • No interference with daily life	• Experiences multiple minor symptoms^1^ • Some improvement with behavior changes^2^ • Some interference with daily life	• Experiences many minor symptoms or any major symptom^1^ • No improvement with behavior changes^2^ • Interferes or limits daily life
Dumping - GI	• No symptoms^3^ • No interference with daily life	• Several symptoms^3^ • Some improvement with behavior changes^2^ • Some interference with daily life	• Many symptoms^3^ • No improvement with behavior changes^2^ • Interferes or limits daily life
Pain	• Mild • No interference with daily life	• Moderate • Some interference with daily life	• Severe • Interferes or limits daily life
Nausea	• No or little nausea • No interference with daily life	• Occasional episodes of no consequence • Responds to medication • Some interference with daily life	• Frequent episodes • Does not respond to medicine • Interferes or limits daily life
Dyspnea	• No symptoms • No interference with daily life	• With exercise or intermittent • Some interference with daily life	• Interferes or limits daily activities
Aspiration	• No aspiration • No noticeable aspiration • No interference with daily life	• Aspiration present • Symptoms are mild • No interference with daily life	• Results in dyspnea or other more severe symptoms • Interrupts or limits daily activities

^**1**^Dumping-Generalized symptoms include: Becoming pale, having a weak pulse or a very low blood pressure, fainting/loss of consciousness/ passing out, dizziness, weakness, exhaustion/desire to lie down due to weakness, sleepiness/drowsiness, palpitations, and headache). ^2^Behavior change includes avoiding sugar or carbohydrates, taking medication, or eating 5–6 meals a day instead of 3 meals a day. ^3^Dumping-GI symptoms include abdominal fullness/abnormal collection of gas in the abdomen, rumbling sound from your stomach or intestines, and diarrhea. Note. Each bullet should be interpreted as “or,” meaning that patients do not have to meet all conditions to be categorized for a certain performance category. If any one condition described in a bullet applies, then the patient is in that performance category.

**Table 2:** Internal consistency estimates comparison for the symptom domains

Domains	Including core and contextual items for scoring	Including only the core items for scoring (frequency, severity, and interference)	Including only the two core items for scoring (severity and interference)
Dysphagia	0.91	0.89	
Heartburn	0.85	0.93	
Regurgitation	0.77	0.83	
Dumping - generalized	0.86	0.87	
Dumping - GI	0.57	0.55	0.68
Pain	0.93	0.93	
Nausea	0.90	0.90	
Dyspnea^a^	-	-	-
Aspiration^a^	-	-	-

^a^ Existing data for dyspnea and aspiration domains were based on single items.

**Table 3:** The final cut scores for good/moderate and moderate/poor performance and standard errors in parentheses

Domains	Good/Moderate	Moderate/Poor	Number of panelists
Dysphagia	21.3 (4.1)	47.5 (2.7)	4
Heartburn	35.0 (1.5)	52.8 (3.8)	4
Regurgitation	27.7 (0.9)	57.8 (1.1)	5
Dumping - generalized	29.2 (5.9)	54.7 (2.9)	3
Dumping - GI	26.7 (2.2)	50.0 (0)	3
Pain	30.7 (3.3)	71.3 (3.7)	5
Nausea	28.2 (1.9)	52.0 (1.3)	5
Dyspnea	27.5 (0)	54.2 (0)	3
Aspiration	29.2 (0)	50.0 (0)	3

Note. Dumping-GI is currently scored with severity and interference items currently, and the cut scores are also based on the two items. All the other domains are scored using frequency, severity, and interference items.

**Table 4:** Panelists’ evaluation of the standard setting process

	Strongly agree	Agree	Disagree/ Strongly Disagree
1. I understood the purpose of the standard setting exercise.	62.5%	37.5%	0%
2. The instructions and explanations provided by the facilitator were clear.	25%	62.5%	12.5%
3. The training on the standard setting method gave me the information I needed to complete my assignment.	25%	62.5%	12.5%
4. The Performance Descriptions that were developed prior to the meeting were accurate.	25%	62.5%	12.5%
5. I understood the concept of the borderline patient.	37.5%	62.5%	0%
6. The Performance Descriptions helped me determine how to rate each item.	25%	62.5%	12.5%
7. It was beneficial to have an opportunity for discussion and to review feedback.	75%	25%	0%
8. The opportunity to provide a second round of ratings (i.e., Round 2) helped me feel more confident about my final ratings.	87.5%	12.5%	0%
9. I felt engaged in the process.	87.5%	12.5%	0%
10. I felt comfortable sharing my ideas with the other panelists during the discussions.	100%	0%	0%
11. I am confident this standard setting process will produce fair cut scores.	62.5%	37.5%	0%
12. I would be comfortable defending this process to my peers.	50%	50%	0%
	**Very influential**	**Somewhat influential**	**Not influential**
13. My perception of the severity of symptoms that the items were measuring	87.5%	12.5%	0%
14. The Performance Descriptions	37.5%	50%	12.5%
15. The average ratings of other panelists	25%	62.5%	12.5%
16. Group discussion after Round 1	50%	50%	0%
17. My experience with patients	75%	25%	0%
	**Very useful**	**Somewhat useful**	**Not useful**
18. Going through PowerPoint training slides prior to beginning the actual rating task	25%	75%	0%
19. Referencing the Performance Descriptions	62.5%	37.5%	0%
20. Group discussion after Round 1	100%	0%	0%
	**Too much time**	**About right**	**Too little time**
21. Reviewing the Performance Descriptions	12.5%	87.5%	0%
22. Round 1 of the rating task	0%	87.5%	12.5%
23. Group discussion after Round 1 to achieve consensus	12.5%	75%	0%

Appendix 2 shows the item-level cut scores given by each panelist, as well as panelist-level cut scores for each domain. Table 3 shows the final cut scores and their SEs. The numbers before the parentheses represent the cut scores for each specified category. For example, in dysphagia, a cut score of 21.3 (with a SE of 4.1) distinguishes good performance from moderate, while a score of 47.5 (with a standard error of 2.7) differentiates moderate performance from poor. Accordingly, 0 ≤ dysphagia score ≤ 21.3 signifies good performance, 21.3 < dysphagia score ≤ 47.5 moderate, and a score > 47.5 reflects poor performance. When incorporating the SE into our interpretation, the range defined by 21.3 ± 4.1 in dysphagia creates a zone of uncertainty, highlighting the area where the true cut score likely falls. Dyspnea and aspiration cut scores had SEs of zero, because panelists reached a perfect consensus

### Evaluation of the standard setting process

Eight panelists (all MDs) returned the evaluation surveys. Three were female. Two were between age 30–39; two age 40–49; two age 50–59; and one ≥ 60. Four (50%) were in thoracic surgery; three (37.5%) general surgery; one (12.5%) physical medicine and rehabilitation; and one (12.5%) endocrinology. Five (62.5%) had > 10 years of experience caring for UDD patients, and three (37.5%) 1–5 years.

The panel (100%) agreed that they understood the purpose of the study (Table 4); the training on the method gave them the information they needed to complete their assignments (87.5%), and that they understood the concept of the borderline patient (100%). One noted that reviewing the concept of the borderline patients was helpful. All expressed it was beneficial to discuss before Round 2. All reported feeling confident that the standard setting process would provide fair cut scores.

## Discussion

In this study, we achieved two goals, (1) identifying cut scores to triage patients, and (2) testing feasibility of virtual standard setting. In general, the discussion between the rounds was deemed very important in clarifying their task, thinking about their patients, and reaching consensus. Panelists expressed high level of faith that the cut scores would yield appropriate care pathways and felt the process of virtual standard setting as acceptable. In addition, we were able to modify the scoring algorithm to be based upon three core items, which was found to be reliable, easier to understand, and aligned better with how panelists understood the patients’ experiences of symptoms.

In this study, we derived the cut scores for nine domains in the UDD Tool™. Compared to the good/moderate thresholds in the previous standard setting [3], which were in the range of 7.2 to 20.8 on a 0-100 scale, the good/moderate thresholds in the current study ranged from 21.3 to 35.0 across domains. The moderate/poor threshold in the previous standard setting ranged from 37.9 to 64.3, which was slightly lower but similar to in the current study 47.5 to 71.3. In this research, we made an extra effort in the training, rating, and post-Round 1 discussion sessions to clarify the concept of borderline patients, highlighting their position as the lowest performers within a category, distinct from average performance. This focus on estimating responses of patients on the verge of transitioning to a poorer category likely influenced the elevation of cut scores, moving away from assessments of typical patient responses to those on the cusp of a lower classification. Furthermore, this study excluded contextual items from the scoring process, a change from our previous methodology. This modification in the scoring approach is also a likely factor in the observed changes in cut scores.

The post-Round 1 discussion proved beneficial. Panelists believed they comprehended the rating task, yet through dialogue, they often realized that they were not considering borderline patients. Sometimes, it was valuable to clarify that, beyond borderline moderate symptoms, they would want medical attention. These discussions improved understanding, refining the final thresholds to more accurately represent the borderline definition.

The virtual meeting was well-received by the panelists. In the earlier paper-based standard setting study [3], wherein panelists manually recorded their ratings on paper (while other standard setting conditions largely remained consistent), 18% of the panelists lacked confidence in the fairness of the cut score production process, and 27% did not feel comfortable defending this process to their peers. In the current study, however, every panelist reported feeling comfortable and confident with the process.

Panelists noted that most symptoms should be interpreted temporally. Relatedly, they stressed the significance of knowing both the absolute performance (good, moderate, and poor) and the normative context of where a patient stands concerning other patients at a specific timepoint post-surgery. Some panelists alluded to responses to certain items important enough to override the decision based on cut scores. For example, panelists noted that problem swallowing liquids would automatically place a patient in the red zone. A future study may incorporate such decision into the scoring algorithm to make use of both the cut scores and the key contextual items when determining clinical action.

Panelists understood the interrelation between patients’ perceived global physical and mental health measured by the PROMIS Global Health, disease status and their symptom perception measured by the UDD tool. They shared that these measures should be interpreted collectively, emphasizing their interconnectedness. Furthermore, Panelists identified domains that cannot be interpreted independently in isolation, such as aspiration, regurgitation, and heartburn; as well as dumping-generalized and dumping-GI.

There are some limitations in this study. We endeavored to conduct groups of 6–8 panelists. However, due to challenges with scheduling practicing physcians, mostly surgeons, the rating exercises consisted of five or fewer panelists per domain. In addition, panelists commented that the cut scores that they were setting for post-esophagectomy patients may not be applicable for all UDD patients (e.g., foregut surgery). Our cut scores have not been validated with patient groups yet, a step that should be undertaken in future research. Lastly, dumping domains were revised to exclude items deemed irrelevant to the content, guided by the insights from this study. Future research may involve assembling experts in the dumping symptoms to assess how well the cut scores align with their categorizations. Furthermore, we adjusted the scoring for the dumping-GI domain to include only two core items, rather than three, a decision based on internal consistency estimates from retrospective data. Given the revised content for the dumping-GI domain at the end of this study, upcoming data collection offers a chance to reassess the appropriateness of using two versus three core items, equipped with new estimates of internal consistency.

## Conclusion

The virtual standard setting process successfully updated cut scores for symptom domains in the UDD Tool™. Panelists were comfortable defending the virtual standard setting process and confident that the methodology would yield valid cut scores. The current study supported score reporting based on three core items: Symptom frequency, symptom severity, and the impact on usual or daily activities. The UDD tool has multiple contextual items that were used for scoring previously but now excluded from scoring, which could play a critical role in clinical decision-making. To address this gap, ongoing research is focused on understanding physician preferences regarding how these contextual items should be presented, including the use of visual aids to facilitate interpretation. These insights will be particularly valuable for developing patient-facing reports in the App or patient portal and creating dashboards tailored for physicians. Such developments are expected to significantly improve the usability of the information, making it more accessible and actionable for both patients and healthcare providers, thereby enhancing the overall effectiveness of care management strategies.

### Electronic supplementary material

Below is the link to the electronic supplementary material.

Supplementary Material 1

Supplementary Material 2

## Data Availability

The datasets generated and/or analyzed during the current study are available in Appendix.

## Abbreviations

UDD: Upper Digestive Disease
SME: Subject matter expert
PRO: Patient-reported outcome
PROM: Patient-reported outcome measure
Dumping-GI: Dumping-Gastrointestinal
