# Supplementary material for: PRO-Angoff method for remote standard setting: establishing clinical thresholds for the upper digestive disease tool
Source: J Patient Rep Outcomes. 2024 Mar 12;8:30. doi: 10.1186/s41687-024-00707-x (PMC10933216; doi:10.1186/s41687-024-00707-x)
Supplement: Supplementary file 1 — Supplementary Material 1 [file 41687_2024_707_MOESM1_ESM.docx]

Appendix 1. Core items and contextual items

1. Pain

| Scored items |  |
| --- | --- |
| Thinking about the past 30 days, mark any locations from the list below where you have experienced pain from your esophagus diagnosis or surgery. (Mark all that apply) | “No pain” 🡪 domain score=0 |
| 1.In the past 30 days, on a scale of 0 to 10, how would you rate the severity of your pain from your esophagus diagnosis or surgery with 0 being “Not at all severe” and 10 being “Very severe”? |  |
| 2. In the past 30 days, how often did you experience pain from your esophagus diagnosis or surgery in the locations you marked above in question 13? |  |
| 3.In the past 30 days, how much has pain interfered with your usual or daily life? |  |
| Contextual information |  |
| Months passed since surgery | We collect surgery date from another database but not from questionnaire |
| 1.Frequency of taking pain medication (I have not taken any pain medication, weekly, daily, all of the time) |  |
| 2.If weekly, how many times per week do you take pain med? |  |
| 3.If daily, how many times per day do you take the pain med? |  |

1. Dysphagia

| Items that contribute to scoring |  |
| --- | --- |
| In the past 30 days, did you try to eat solid foods? | If “No, told to avoid solid foods” 🡪 Missing domain score |
| 1.How often have you had difficulty swallowing? | Never🡪 Score of 0 |
| 2. In the past 30 days, how much has your difficulty swallowing interfered with your usual or daily life? |  |
| 3. In the past 30 days, on a scale of 0 to 10, how would you rate the severity of your trouble swallowing with 0 being “Not at all severe” and 10 being “Very severe”? |  |
| Contextual information |  |
| 1.In the past 30 days, which one of the following best describes your swallowing in general? (able to eat or drink anything to extreme difficulty) |  |
| 2.On the days that you had difficulty swallowing, what was the frequency? |  |
| 3.In the past 30 days, did you have difficulty swallowing cold liquids? |  |
| 4.In the past 30 days, did you have difficulty swallowing warm liquids? |  |
| 5.In the past 30 days, did you have difficulty swallowing solid foods, such as XXX? |  |
| 6.In the past 30 days, have you had food (not medications) stick in your esophagus/swallowing tube? |  |
| 7.In the past 30 days, when you had solid food get stuck in your esophagus/swallowing tube, how long was it stuck? |  |
| 8.In the past 30 days, when you had solid food get stuck in your esophagus/swallowing tube, did you have problems swallowing liquids after solid food was stuck? |  |
| 9.In the past 30 days, when you had solid food get stuck in your esophagus/swallowing tube, did it make you vomit? |  |
| 10.In the past 30 days, how many times did you eat each day on average? Count both meals and snacks |  |
| 11.In the past 30 days, what was the average portion size (how much food you ate at one time) for each meal or snack? |  |
| 12.In the past 30 days, how many minutes did it take you to eat an average meal or snack? |  |
| 13.In the past 30 days, if you take pills, have you had pills stick in your esophagus/swallowing tube? |  |
| 14.In the past 30 days, which of the following best describes your pain or discomfort when swallowing? |  |
| 15.Have you ever had a dilation (stretching) procedure of the esophagus/swallowing tube? |  |

*Lactose intolerance (Not scored)

| 1.In the past 30 days, did you have loose bowel movements when you drank milk or ate milk products? |  |
| --- | --- |
| 2.In the past 30 days, did you have abdominal pain or cramping when you drank milk or ate milk products? |  |

1. Heartburn

| Items that contribute to scoring |  |
| --- | --- |
| 1.How often do you experience heartburn? | “Never” – score of 0 |
| 2. In the past 30 days, how much has heartburn interfered with your usual or daily life? |  |
| 3. In the past 30 days, on a scale of 0 to 10, how would you rate the severity of your heartburn with 0 being “Not at all severe” and 10 being “Very severe”? |  |
| Contextual information |  |
| 1.In the past 30 days, have you taken antacids to prevent or manage heartburn? (Examples: Amphojel, Alternagel, Gaviscon, Maalox, Mylanta, Riopan, Rolaids, Tums used as an antacid) |  |
| 2.In the past 30 days, has your heartburn awakened you at night? |  |
| 3.In the past 30 days, has your heartburn often traveled up toward your neck? |  |

4. Regurgitation

| Items that contribute to scoring |  |
| --- | --- |
| 1.How often have you had any regurgitation? | “Never” – score of 0 |
| 2. In the past 30 days, on a scale of 0 to 10, how would you rate the severity of your regurgitation with 0 being “Not at all severe” and 10 being “Very severe”? |  |
| 3. In the past 30 days, how much has regurgitation interfered with your usual or daily life? |  |
| Contextual information |  |
| 1.Are you able to burp? |  |
| 2.In the past 30 days, did you stop eating within 2 hours of going to bed on most nights? |  |
| 3.In the past 30 days, did you elevate your head while sleeping? |  |
| 4.Mark any types of regurgitation that you experienced: Acid, saliva, food |  |
| 5.Did you cough up liquids from your throat? |  |
| 6. Have you taken prescription or over the counter medication to prevent or manage regurgitation? |  |

1. Nausea

| Items that contribute to scoring |  |
| --- | --- |
| 1.How often did you experience nausea? | “Never” – score of 0 |
| 2. In the past 30 days, on a scale of 0 to 10, how would you rate the severity of your nausea with 0 being “Not at all severe” and 10 being “Very severe”? |  |
| 3. In the past 30 days, how much has nausea interfered with your usual or daily life? |  |

1. Dumping-Generalized

| Items that contribute to scoring |  |
| --- | --- |
| Becoming pale, having a weak pulse, or a very low blood pressure | If a person marked **No** or **Unsure** or skipped items on the left column, then the domain score is **0**.  If a person responded “**Yes**” to at least one of items in the left column, then scores are based on the **next 3 items**. |
| Fainting, loss of consciousness, passing out |  |
| Dizziness |  |
| Breathlessness, shortness of breath |  |
| weakness, exhaustion, desire to lie down due to weakness |  |
| sleepiness, drowsiness |  |
| abnormal or rapidly beating heart (palpitations) |  |
| Headaches |  |
| Sweating, feeling warmth, clammy |  |
| 1.In the past 30 days, how often did you experience the dumping-generalized (whole body) symptoms you marked above? |  |
| 2.In the past 30 days, on a scale of 0 to 10, how would you rate the severity of your dumping- generalized (whole body) symptoms with 0 being “Not at all severe” and 10 being “Very severe”? |  |
| 3.In the past 30 days, how much have dumping- generalized (whole body) symptoms interfered with your usual or daily life? |  |
| Contextual information |  |
| 1.When you experienced the dumping- generalized (whole body) symptoms you marked above, was it with each meal? |  |

1. Dumping-GI

| Items that contribute to scoring |  |
| --- | --- |
| Nausea or feeling like wanting to throw up | If a person marked **No** or **Unsure** or skipped items on the left column, then the domain score is **0**.  If a person responded “**Yes**” to at least one of these items, then scores are based on the **next 2 items**. |
| Abdominal fullness, abnormal collection of gas in the abdomen |  |
| Rumbling sound from your stomach or intestines |  |
| Diarrhea |  |
| 1.In the past 30 days, on a scale of 0 to 10, how would you rate the severity of your dumping- gastrointestinal symptoms with 0 being “Not at all severe” and 10 being “Very severe”? |  |
| 2.In the past 30 days, how much have dumping- gastrointestinal symptoms interfered with your usual or daily life? |  |
| Contextual information |  |
| 1.In the past 30 days, how often did you experience the dumping-gastrointestinal symptoms you marked above? | Tentatively contextual |
| 2.When you experienced the dumping- gastrointestinal symptoms you marked above, was it with each meal? |  |
| 3.Thinking of both dumping-generalized and dumping-gastrointestinal, do any of the following improve your “dumping syndrome” symptoms? (Mark all that apply) |  |
| 4. Have you taken any medications in the past 30 days for “dumping syndrome”: Tolbutamide, Propranolol, Cyproheptadine, Methysergide maleate, Verapamil, Acarbose, Octreotide or other treatments specifically for dumping? |  |

1. Breathlessness

| Items that contribute to scoring |  |
| --- | --- |
| 1.How often did you experience breathlessness? | “Never” – score of 0 |
| 2. In the past 30 days, on a scale of 0 to 10, how would you rate the severity of your breathlessness with 0 being “Not at all severe” and 10 being “Very severe”? |  |
| 3. In the past 30 days, how much has breathlessness interfered with your usual or daily life? |  |

1. Aspiration

| Items that contribute to scoring |  |
| --- | --- |
| 1.How often did you experience aspiration? | “Never” – score of 0 |
| 2. In the past 30 days, on a scale of 0 to 10, how would you rate the severity of your aspiration with 0 being “Not at all severe” and 10 being “Very severe”? |  |
| 3. In the past 30 days, how much has aspiration interfered with your usual or daily life? |  |
| Contextual information |  |
| 1. In the past 30 days, have you experienced an infection in your lungs (ex. Common cold, pneumonia, bronchitis, other respiratory infection, cough producing phlegm)? |  |

Appendix 2. Item-level cut scores per panelist as well as panelist-level cut scores on a 0-100 reporting scale

|  | **Frequency** | **Severity** | **Interference** | **Panelist-level cut score on 0-100 reporting scale** |
| --- | --- | --- | --- | --- |
| **Dysphagia Good/Moderate cut score** | | | | |
| Panelist 1 | 1.5 | 1.5 | 1 | 20.83 |
| Panelist 2 | 2 | 2.5 | 2 | 31.67 |
| Panelist 3 | 1.5 | 1 | 1.5 | 20.83 |
| Panelist 4 | 1 | 1 | 0 | 11.67 |
|  |  |  |  |  |
| **Dysphagia Moderate/Poor cut score** | | | | |
| Panelist 1 | 2.5 | 4.5 | 4 | 49.17 |
| Panelist 2 | 2.5 | 5 | 5 | 54.17 |
| Panelist 3 | 2 | 4.5 | 4 | 45 |
| Panelist 4 | 3 | 2 | 3 | 41.67 |
|  |  |  |  |  |
| **Heartburn** **Good/Moderate cut score** | | | | |
| Panelist 1 | 2 | 3 | 2 | 33.33 |
| Panelist 2 | 2 | 3 | 3 | 36.67 |
| Panelist 3 | 2 | 2.5 | 2 | 31.67 |
| Panelist 4 | 3 | 2.5 | 1.5 | 38.33 |
|  |  |  |  |  |
| **Heartburn Moderate/Poor cut score** | | | | |
| Panelist 1 | 3 | 4.5 | 4.5 | 55 |
| Panelist 2 | 3 | 5 | 4 | 55 |
| Panelist 3 | 3.5 | 5 | 4 | 59.17 |
| Panelist 4 | 3 | 3 | 2 | 45.83 |
|  |  |  |  |  |
| **Regurgitation Good/Moderate cut score** | | | | |
| Panelist 1 | 2 | 2.5 | 1 | 28.33 |
| Panelist 2 | 2 | 2 | 1.5 | 28.33 |
| Panelist 3 | 2 | 1.5 | 1 | 25 |
| Panelist 4 | 2 | 1.5 | 1.5 | 26.67 |
| Panelist 5 | 2 | 2.5 | 1.5 | 30 |
|  |  |  |  |  |
| **Regurgitation Moderate/Poor cut score** | | | | |
| Panelist 1 | 2.5 | 6 | 6 | 60.83 |
| Panelist 2 | 3 | 5 | 5 | 58.33 |
| Panelist 3 | 2.5 | 5 | 5 | 54.17 |
| Panelist 4 | 2.5 | 5.5 | 5.5 | 57.5 |
| Panelist 5 | 3 | 5 | 5 | 58.33 |
|  |  |  |  |  |
| **Dumping-generalized Good/Moderate cut score** | | | | |
| Panelist 1 | 0.5 | 2 | 2 | 17.5 |
| Panelist 2 | 2 | 3 | 3 | 36.67 |
| Panelist 3 | 2 | 2 | 3 | 33.33 |
|  |  |  |  |  |
| **Dumping-generalized Moderate/Poor cut score** | | | | |
| Panelist 1 | 2 | 5 | 5 | 50 |
| Panelist 2 | 2.5 | 5 | 5 | 54.17 |
| Panelist 3 | 3 | 6 | 4.5 | 60 |
|  |  |  |  |  |
| **Dumping-GI Good/Moderate cut score** | | | | |
| Panelist 1 | a | 2.5 | 3 | 27.5 |
| Panelist 2 | a | 2 | 2.5 | 22.5 |
| Panelist 3 | a | 2.5 | 3.5 | 30 |
|  |  |  |  |  |
| **Dumping-GI Moderate/Poor cut score** | | | | |
| Panelist 1 | a | 5 | 5 | 50 |
| Panelist 2 | a | 5 | 5 | 50 |
| Panelist 3 | a | 6 | 4 | 50 |
|  |  |  |  |  |
| **Pain Good/Moderate cut score** | | | | |
| Panelist 1 | 2 | 3 | 3 | 36.67 |
| Panelist 2 | 2 | 2 | 3 | 33.33 |
| Panelist 3 | 1.5 | 2 | 1.5 | 24.17 |
| Panelist 4 | 1 | 2 | 2 | 21.67 |
| Panelist 5 | 2.5 | 2 | 3 | 37.5 |
|  |  |  |  |  |
| **Pain Moderate/Poor cut score** | | | | |
| Panelist 1 | 3 | 7 | 6 | 68.33 |
| Panelist 2 | 5 | 6 | 6 | 81.67 |
| Panelist 3 | 3.5 | 5 | 5 | 62.5 |
| Panelist 4 | 3.5 | 5.5 | 5.5 | 65.83 |
| Panelist 5 | 5 | 6 | 5 | 78.33 |
|  |  |  |  |  |
| **Nausea Good/Moderate cut score** | | | | |
| Panelist 1 | 2 | 3 | 2 | 33.33 |
| Panelist 2 | 1.5 | 3 | 2 | 29.17 |
| Panelist 3 | 1 | 2 | 2 | 21.67 |
| Panelist 4 | 1.5 | 2.5 | 2 | 27.5 |
| Panelist 5 | 1.5 | 3 | 2 | 29.17 |
|  |  |  |  |  |
| **Nausea Moderate/Poor cut score** | | | | |
| Panelist 1 | 2 | 5 | 5 | 50.0 |
| Panelist 2 | 2 | 5 | 5 | 50.0 |
| Panelist 3 | 2 | 6 | 6 | 56.67 |
| Panelist 4 | 2 | 5.5 | 5.5 | 53.33 |
| Panelist 5 | 2 | 5 | 5 | 50.0 |
|  |  |  |  |  |
| **Dyspnea Good/Moderate cut score** | | | | |
| Panelist 1 | 1.5 | 1.5 | 3 | 27.5 |
| Panelist 2 | 1.5 | 1.5 | 3 | 27.5 |
| Panelist 3 | 1.5 | 1.5 | 3 | 27.5 |
|  |  |  |  |  |
| **Dyspnea Moderate/Poor cut score** | | | | |
| Panelist 1 | 2.5 | 5 | 5 | 54.2 |
| Panelist 2 | 2.5 | 5 | 5 | 54.2 |
| Panelist 3 | 2.5 | 5 | 5 | 54.2 |
|  |  |  |  |  |
| **Aspiration Good/Moderate cut score** | | | | |
| Panelist 1 | 0.5 | 2 | 5.5 | 29.2 |
| Panelist 2 | 0.5 | 2 | 5.5 | 29.2 |
| Panelist 3 | 0.5 | 2 | 5.5 | 29.2 |
|  |  |  |  |  |
| **Aspiration Moderate/Poor cut score** | | | | |
| Panelist 1 | 2 | 3.5 | 6.5 | 50.0 |
| Panelist 2 | 2 | 3.5 | 6.5 | 50.0 |
| Panelist 3 | 2 | 3.5 | 6.5 | 50.0 |

Note. The frequency item is measured on a 0-4 ordinal scale, and severity and interference items on a 0-10 numerical rating scale. The three items are weighted equally so we multiply the frequency item score by 2.5. The total raw score ranges from 0-30. The dumping-GI is based on only the severity and interference items currently, thus the total raw score ranges from 0-20. The conversion to 0-100 scale is computed by multiplying the raw scores by 100 divided by the maximum total raw score possible (20 for dumping-GI and 30 for all other symptom domains).

a. We obtained panelists’ cut scores for dumping-GI frequency item, with scores of 2, 1.5, and 2 for borderline good and scores of 3, 3, and 4 for borderline moderate. However, these scores have not been factored into the current cut score calculations due to the observation that including this item resulted in reduced internal consistency estimates for this domain.
